# National Diabetes Month — November 2014

**Published:** 2014-11-21

November is National Diabetes Month. In the United States, about 29 million persons have diabetes, including 8 million who do not know they have it (1). In addition, about 86 million adults have prediabetes, putting them at increased risk for developing type 2 diabetes, heart disease, and stroke, and only 11% know they have it (1,2). However, persons with diabetes can take steps to control the disease and prevent complications, and those with prediabetes can prevent or delay the onset of type 2 diabetes through weight loss and physical activity (3). A recent study showed that after decades of continued growth in the rate of new cases of diagnosed diabetes, the rate of increase in new cases might have leveled off (4).

CDC and its partners support programs to prevent and control diabetes. CDC’s National Diabetes Prevention Program promotes community-based lifestyle change programs for persons at risk for type 2 diabetes throughout the United States (5). CDC’s Native Diabetes Wellness Program supports health promotion and prevention of type 2 diabetes in American Indian/Alaska Native communities. The National Diabetes Education Program, jointly sponsored by CDC and the National Institutes of Health, provides tools and resources to help organizations and individuals address diabetes in their communities, health care practices, and businesses.
